# Developing and adapting two electronic‐rehabilitation programmes for persistent knee pain

**DOI:** 10.1002/msc.1812

**Published:** 2023-08-25

**Authors:** Dawn Groves‐Williams, Elizabeth C. Lavender, Christine Comer, Mark Conner, Rachel K. Nelligan, Kim L. Bennell, Sarah R. Kingsbury, Philip G. Conaghan, Gretl A. McHugh

**Affiliations:** ^1^ Clinical Trials Research Unit University of Leeds Leeds UK; ^2^ School of Healthcare University of Leeds Leeds UK; ^3^ Musculoskeletal and Rehabilitation Service Leeds Community Healthcare NHS Trust Leeds UK; ^4^ School of Psychology University of Leeds Leeds UK; ^5^ Department of Physiotherapy The University of Melbourne Centre for Health Exercise and Sports Medicine Victoria Australia; ^6^ Leeds Institute of Rheumatic and Musculoskeletal Medicine University of Leeds Leeds UK

**Keywords:** digital health, exercise, knee osteoarthritis, pain, rehabilitation

## 1 INTRODUCTION

Persistent knee pain affects one in four people, limiting physical function and mobility and affecting the quality of life (Bindawas et al., 2015; Jinks et al., 2002; Nguyen et al., 2011). The leading cause of persistent knee pain in those over 45 years of age is osteoarthritis (OA).

Painful knee OA can be improved by tackling some of the risk factors such as obesity and physical inactivity (Bannuru et al., 2019). Guidelines from the National Institute for Health and Care Excellence (NICE) recommend information and exercise for the management of knee OA (NICE, 2022). Physiotherapists are key providers of exercise therapy, support and guidance. However, the demand for physiotherapy services continues to be high and in some areas of the United Kingdom (UK) the wait for treatment is long. There is evidence from a systematic review that longer waits can negatively impact pain, disability and quality of life and leads to higher healthcare use and costs (Deslauriers et al., 2021). Our public involvement activities with people living with persistent knee pain highlighted two concerns: difficulty accessing physiotherapy appointments due to mobility and transport issues, also highlighted in the literature (Tan et al., 2017), and a short duration of physiotherapy support and desire for more exercise and self‐management guidance.

People living with knee OA often seek information from the Internet (Jellison et al., 2018), yet the sources and quality of information are variable. Digital health interventions are an alternative approach to clinic‐based delivery but still offer the opportunity to be individualised. Some have a strong evidence‐base and are seen as an accessible low‐cost alternative to managing health conditions (Centre for Policy on Ageing, 2014; Morris et al., 2014; Rogers et al., 2017). An Australian study tested one such intervention for persistent knee pain, involving an internet‐delivered physiotherapist‐prescribed home exercise (one‐to‐one) programme combined with self‐directed pain‐coping skills training (Bennell et al., 2017). The intervention resulted in clinically relevant improvements in pain and function (Bennell et al., 2017). A qualitative evaluation of this programme found that using the Internet/Skype as a service delivery model for exercise management was a positive experience for both patients and physiotherapists and one which offered a more ‘personalised’ service than traditional clinic‐based care (Hinman et al., 2017). Another Australian electronic‐rehabilitation (e‐rehab) programme, this time involving a fully self‐directed 6‐month web‐based exercise intervention without any physiotherapist contact (My Knee Exercise) was also effective in improving knee pain and physical function (Nelligan et al., 2021).

To address issues highlighted by patients and overstretched physiotherapy services, we set out to investigate whether digitally delivered physiotherapy services and exercise treatment are a viable solution for managing persistent knee pain. This paper reports on the development of a group e‐rehab programme (Group E‐Rehab) and adaptation of the Australian My Knee Exercise programme (https://mykneeexercise.org.au) (Nelligan et al., 2021) to My Knee UK. A protocol of the randomised feasibility trial for the programmes is published (Groves‐Williams et al., 2022).

## 2 METHODS

**FIGURE 1:**
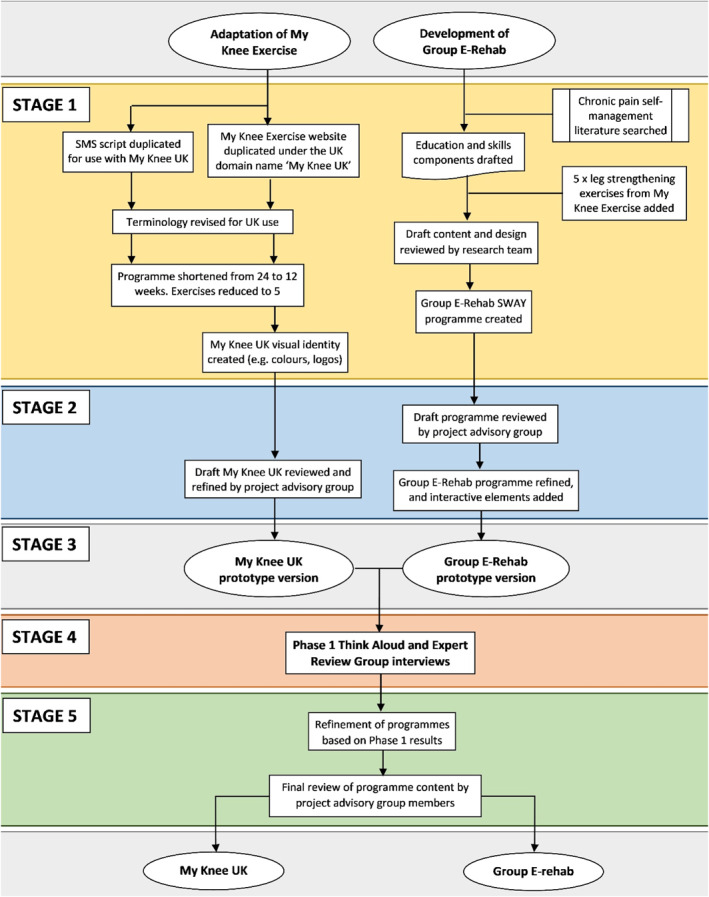
Intervention Development Process. *Programme Development*: *Stage 1* ‐ Draft development and adaptation of the interventions by the research team; *Stage 2*: Review and refinements to programme designs by project advisory group; *Creation of prototypes*: *Stage 3*; *User‐testing and refinement*: *Stage 4* ‐ review of prototypes by think‐aloud and expert review group interviews; *Final Review*: *Stage 5* ‐ Further refinements reviewed by project advisory group and both programmes finalised.

A summary of the staged development of Group E‐Rehab and adaptation of My Knee UK is provided in Figure 1. Table 1 provides an overview of the two programmes with a detailed description published in the study protocol (Groves‐Williams et al., 2022). Patient and Public Involvement (PPI) was integral throughout the study.

**TABLE 1:** Description of Group E‐rehab and My Knee United Kingdom (UK).

Programme	Overview	Content
Group E‐Rehab	12‐week internet delivered programme 7 remotely delivered physiotherapy‐led group sessions (demonstration and observation of exercises; adaption of exercises as required); Self‐directed strengthening exercises; Information resources using Microsoft Sway.	Interlinked components Guided lower limb strengthening exercise programmes; Knowledge building (6 ‘Sway’ sessions: Pain and the knee joint; Physical activity; Goal setting; Pacing skills; Communication and emotional wellbeing; Staying healthy); Developing skills (e.g. goal setting).
My Knee UK	12‐week self‐directed internet delivered programme Education and skills development; Progressive lower limb strengthening exercises; Physical activity tracking with aim of increasing physical activity; Automated text messages designed to prompt activity, encourage adherence, and help participants identify barriers to engagement (Nelligan et al., 2019).	Introduction (to using the programme; help information); My Knee Education (information about exercise/knee pain/knee OA); My Knee Strength (e.g. exercise programme); My Knee Activity (e.g. physical activity plan); My Knee Tools (all resources in one area of website).

### 2.1 Stages 1 and 2: Programme development

For the development of Group E‐Rehab, we used the existing evidence base, including components from a developed internet‐delivered exercise programme (Bennell et al., 2017), resources for guided self‐management of OA (Anderson et al., 2021), and the evidence‐base for MSK pain management (Main & Spanswick, 2000; Stones & Cole, 2014; Torrance et al., 2011).

Our approach to developing My Knee UK involved continuous input from key stakeholders and engagement with the developers of My Knee Exercise. Guidance on adapting interventions to new contexts has since recommended this approach (Moore et al., 2021). For the initial adaptation, the My Knee Exercise website was duplicated under the UK domain name My Knee UK, and the language and terminology were modified for UK use with format changes. The original behaviour‐change text message script was also duplicated and modified for UK use (Nelligan et al., 2019). The UK version of the programme and exercises were shortened from 24‐week to 12‐week to improve adherence (Eckard et al., 2015; Henry et al., 1999).

### 2.2 Stage 3: Creation of the prototypes

Following recommendations for the design of the prototype (Hawkins et al., 2017; O’Cathain et al., 2019), our two e‐rehab programmes were reviewed by the study team and two project advisory group PPI members who live with persistent knee pain. The prototype versions for Group E‐Rehab and My Knee UK were created for user‐testing and refinement.

### 2.3 Stage 4: User‐testing and refinement

A qualitative review of the prototypes of Group E‐Rehab and My Knee UK took place in October 2020. Ethical approval was granted by the West of Scotland Research Ethics Committee 5 (Ref:20/WS/006). This stage involved user‐testing and expert review of both programmes using think‐aloud interviews.

A sample size of 5 and 10 participants is deemed sufficient for think‐aloud interviews (Bishop et al., 2016; Neilson, 1994). Participants recently discharged from an NHS community musculoskeletal clinic aged 45 years and older and with knee OA pain were invited to take part. Of the 140 invitations sent, 22 responded indicating an interest to take part, four declined for personal reasons, four had no knee pain and four were unable to participate via video‐call (necessary due to Covid‐19 restrictions at the time).

The think‐aloud interviews were undertaken by video call using a topic guide focusing on the content, usability, and support mechanisms of both programmes. Participants were asked to speak out loud their thoughts as they worked through the interactive educational sessions and exercise components of Group E‐Rehab and the My Knee UK website in the presence of a researcher. The interviews were recorded, transcribed verbatim and analysed using conventional content analysis with coding categories developed from the interview data (Hsieh & Shannon, 2005).

Expert review was used to complement the think‐aloud interviews and provided additional insight into any potential usability issues (Maramba et al., 2019). Key stakeholders were recruited using snowballing (Green & Aarons, 2011) and included people living with persistent knee pain, physiotherapists, rheumatologists, commissioners of health services and representatives from an arthritis charity. Expert review sessions were undertaken by video‐call and covered elements of the two prototypes relevant to the group's specific expertise; for example, physiotherapists focused on the exercise components and commissioners commented on real‐world application. These sessions were recorded, and content analysed as described above.

### 2.4 Stage 5: Final review

This qualitative review enabled our two programmes to be refined with a final review by PPI members and project advisory group.

## 3 RESULTS

Key findings from the qualitative review (Stage 4) are summarised here.

Twenty‐six people participated. Ten people (6 male, 4 female) living with persistent OA knee pain consented and took part in the think‐aloud interviews. The average age was 68 years (range 54–78 years) with interviews lasting approximately 80 min. Sixteen stakeholders took part in five expert review sessions, including people living with persistent knee pain (*n* = 3), physiotherapists (*n* = 7), rheumatologists (*n* = 2), clinical commissioners (*n* = 2), and representatives from arthritis charity (*n* = 2). On average, each session lasted 60 min.

The common themes which emerged from both the think‐aloud interviews and expert review sessions included accessibility, motivation, and engagement. Table 2 provides an overview of the themes, categories and meaning units from the think‐aloud interviews for both programmes. These themes emerged in relation to the exercise and educational components, self‐management, and format of the intervention.

**TABLE 2:** Themes, categories, and meaning unit from think‐aloud (TA) Interviews.

		My Knee UK	Group E‐Rehab
Theme	Categories	Meaning unit	Meaning unit
Accessibility	Usability	…I'm just trying to quickly scan read it…I'm just more…to be quite honest I am actually struggling with the size of the font ….that's really making me struggle… (*TA002‐female*, *64 years*, *working)*	…the only problem that I can see from this is, there is a lot of information on here [topic goal planning and implementation]…and would people get bored with reading it all after a certain amount of time, I'm not sure. (*TA004‐female*, *57 years*, *working*)
	Appearance	…I like the picture at the top because I like photography, so I look at pictures quite a lot, I like the nature link and the fact that people are walking and cycling, and that's what they want to do with our knees, to get back to that, so I think that's a good hook in for a starting point. (*TA002‐female*, *64 years*, *working*)	I like the pictures, I like the old people and the young people together which, because it affects everybody doesn't it. (*TA004‐female*, *57 years*, *working*)
	Digital capability	Yeah, well maybe, it's somebody that's coming to it new that's never been, used a computer before, which probably with osteoarthritis it's an age thing, if you're getting a lot of older people that have not used computers, maybe it might be better to say have a little that sign that says ‘click on these boxes’…yeah. (*TA007‐male*, *73 years*, *retired*)	I think you might, a lot of elderly people might struggle with it [topic problem solving]. They like to have a bit of paper from the doctor. (*TA003‐male*, *72 years*, *retired*)
	Understanding	…a lot of people don't think they should exercise when they've got knee pain, they think it will make it worse…so people do need educating in that. (*TA004‐female*, *57 years*, *working*)	I think people do want more and more information, but they want accurate evidence‐based information rather than just something they've read from some site…or a friend has told them, so that's good is that. (*TA008‐female*, *68 years*, *working*)
Motivation	Self‐tailoring	…it's all clear what you should do, when you should do it, how you should do it, and then if it's too much to…you know, stop doing it as much and do less. (*TA009‐female*, *54 years*, *working*)	Any information you don't need you can skip, but if you do need it, it's there… *(TA001‐male*, *67 years*, *retired)*
	Feeling supported	…you're doing it by yourself but you're not alone, you know, there is a team. OK they're automated messages that are coming over, but none the less people are showing an interest in you and I think that's crucial… (*TA006‐male*, *78 years*, *retired*)	I like the idea of the one with the physiotherapist [Group E‐rehab] so in a sense I still feel I'm connected to the medical profession while I'm doing it. I'm not just out there on my own. (*TA002‐female*, *64 years*, *working*)
	Interactive	I think that's helpful [exercise logbooks] because it does remind you if you've skipped or you haven't done it. (*TA002‐female*, *64 years*, *working*)	If you fill this questionnaire in about ‘how physically active am I’ [Topic: Self‐assessment]…does it tell you at the end of the questionnaire, how active you are…maybe that could be thought about. You know some people might think they're active and they're not as active as they think. (*TA007‐male*, *73 years*, *retired)*
Engagement	Content	I think that's quite concise isn't it? Yeah, I think there's enough information there for people to have the knowledge that they need without overloading them. *(TA008‐female*, *68 years*, *working)*	It would be a good idea [to send printed copies]…again, if there are more elderly people who are not computer literate that would be supportive. (*TA002‐ female*, *64 years*, *working*)
	Confidence	…if you have a [physical activity] plan and you follow the plan, you're not going to overdo things are you… you feel great when it's done. (*TA007‐male 73 years*, *retired*) “You can help yourself” I think that's a really powerful statement…too many people just think we can go to the doctor and get a pill…and I think, actually what we need to do is go to the doctor and be directed to the right channels to help yourself. *(TA002‐female*, *64 years*, *working)*	I think it's [educational package] is one of the best I've seen of anything like this where it's helping…it's helping you to make decisions on your own future. (*TA006‐male*, *78 years*, *retired*) …if it isn't effective people…after a few weeks people aren't gonna do it are they. (*TA010—male*, *72 years*, *retired*)
	Interactive	…something like this [planning and recording physical activity] makes you think hang on, I need to get back on track. (*TA003‐male*, *72 years*, *retired*)	I don't think [quizzes] should be mandatory. You don't want people thinking “oh I'm not doing that again there's a test every time you do it”. (*TA003‐male*, *72 years*, *retired*)
	Technique	…that's brilliant [exercise videos]…also, I think when you see somebody do it, you…you see the level of energy that's required to do it. (*TA006‐ male*, *78 years*, *retired*)	I think it's a much better idea to have a connection via zoom with a physio because then you feel you're doing the right thing and it's reassuring, and you could ask a question if your technique is right. (*TA002‐female*, *64 years*, *working*)
	Consistency	…decide what you'd like to do…increase your physical activity by setting a goal…keep them on track…yeah. Like you said it's about making time isn't it. Make it easier for you to stick to the plan. *(TA009‐female*, *54 years*, *working)*	…from what I've seen today, if you follow the…the way through, then at some point you should be able to do reasonable exercise for a reasonable amount of time. (*TA006‐ male*, *78 years*, *retired*)

*Exercise component*: The exercise component of both programmes was positively received with favourable reviews of the pictorial presentation, lack of equipment requirements and pointers towards incorporating exercises into activities of daily living. Important elements for participants included options for self‐tailoring muscle‐strengthening exercises, ensuring that exercises (level and number) are challenging but ‘do‐able’, and having an indication of time required to complete the exercises. Confidence building through viewing video‐exercise demonstrations in My Knee UK was helpful for participants to assess their ability to do the exercises. Having input from a physiotherapist and peer support in Group E‐Rehab were considered motivators. However, exercising in a group, even online, was perceived to be intimidating for some.

*Educational component*: Participants commented on the large amount of information provided within both programmes and wanted options to focus on sections of interest and/or relevance to them. Having success stories of people who have benefitted from similar exercise programmes was considered important inclusion in both. The optional interactive elements (e.g. quizzes and self‐assessments) were thought to be motivating; however, if completion was made compulsory, this might be off‐putting for some people.

*Self‐management of persistent OA knee pain*: Encouraging people to set goals and track progress was considered useful; however, participants thought it unnecessary to make these elements mandatory. Logging activity is a motivational tool for goal‐setting and self‐reward. Enabling activity logs to be downloaded or providing paper copies was an important element. Emotional support and wellbeing tips were considered important, but participants did not agree that they were separate topics, which could be overlooked/dismissed. They thought that integrating tips throughout the programmes would lead to better engagement. Support for self‐management was considered essential; however, it was acknowledged that relying solely on automated texts within My Knee UK might not be enough to induce behaviour change.

*The format of intervention*: Two key issues raised were the efficacy of support mechanisms and the digital capacity of users. Group E‐Rehab enables guided group exercise with social interaction facilitated by a physiotherapist, whereas My Knee UK relies on individual's motivation to engage. Expert review participants felt that the group structure might encourage people to sustain engagement over the 12‐week programme. However, the flexibility of My Knee UK might appeal to those constrained by personal or work commitments and those unwilling to engage in group activities.

Digital capability was anticipated to be a significant issue for programme users and was central to the three themes of accessibility, motivation and engagement. Although both programmes were designed to be simple and easy to use, it was highlighted that people may find accessing the programmes or joining group video sessions challenging. Clearer instructions on ‘how to use’ were subsequently developed for both. Digital poverty was highlighted as an issue. Concern was expressed for people without Internet access being able to engage with e‐rehab.

The findings from this qualitative review were used to adapt the two e‐rehab programmes for evaluation in a feasibility trial (Groves‐Williams et al., 2022).

## 4 DISCUSSION

The aim of this study was to develop and adapt for UK use, two e‐rehab programmes for persistent knee pain. The process of development and refinement reported here highlights the importance of user‐testing and expert review by key stakeholders. Using a staged‐approach to developing Group E‐Rehab and adapting My Knee UK enabled us to gain insights from a range of experts and undergo user‐testing by iteratively refining the two programmes prior to feasibility testing. Obtaining user perspectives at the design stages enhances acceptability (Skehon et al., 2017). A recent formal guidance on adapting interventions for use in different contexts supports the approach we used for developing My Knee UK (Moore et al., 2021).

The qualitative review highlighted issues around usability, relevance and format of both programmes. As My Knee UK was being adapted from an Australian website, modifications were needed to ensure it was acceptable to users in the UK. For both programmes, many suggestions were made around improving the visual presentation. These were simple changes but ones which greatly enhanced usability and acceptability.

The Group E‐Rehab programme was designed to have scheduled input from a physiotherapist and group peer support. These were viewed as potential motivators for engagement with the programme. Other research has found that having a ‘human connector’ affiliated with a digital intervention is important (Nelligan et al., 2020). My Knee UK did not include these motivational elements but did allow for greater flexibility with engagement and included the text message programme as a way to try to optimise engagement without a human element. This could stimulate self‐motivation to exercise amongst potential users.

Digital capability was a key concern raised by consumers and stakeholders. As people with knee OA are often older, they are more likely to have low digital literacy (Anderson & Perrin, 2017). A report indicated an increase in Internet usage since the start of the pandemic, but 42% of those 75+years are not using the Internet (Age UK, 2021). Digital literacy is important to consider when developing and implementing digital health interventions, including what support will be provided to those who need additional skills and confidence to use digital health technology. From this qualitative review, findings indicate that both e‐rehab programmes were well‐designed and easy to use.

Limitations of this study included conducting the think‐aloud interviews remotely due to Covid‐19 restrictions, which limited the participation of some people due to digital capability and access. The think‐aloud interviews had to be modified for remote involvement with the participants. This may have restricted the ‘thinking aloud’ process as participants had to rely more on the researcher to take the lead in exploring the components of the programmes. There were also limitations with the diversity of the sample. Holding expert review sessions remotely limited interaction between groups of reviewers. Having more heterogenous groups, including better representation from underserved communities, may have enabled different perspectives to be explored.

## 5 CONCLUSION

The approach to refining and improving accessibility of two e‐rehab programmes for the target population has been described. The input of experts and user reviewers has been key to this process. Group E‐Rehab and My Knee UK offer a potential novel solution to people with persistent knee pain and further research will investigate the effectiveness of both programmes as an alternative to traditional physiotherapy services.

## AUTHOR CONTRIBUTIONS

Funding was secured by Gretl A. McHugh, Philip G. Conaghan, Sarah R. Kingsbury, Kim L. Bennell, Mark Conner and Christine Comer. The Australian SMS Programme and My Knee Exercise were co‐developed by Kim L. Bennell and Rachel K. Nelligan. The qualitative review was developed by Gretl A. McHugh and data were collected and analysed by Elizabeth C. Lavender and Dawn Groves‐Williams. Dawn Groves‐Williams undertook the development and adaptation of the two programmes. Gretl A. McHugh, Dawn Groves‐Williams and Elizabeth C. Lavender drafted the manuscript and all authors contributed to the review of the manuscript and approved the final version.

## CONFLICT OF INTEREST STATEMENT

None declared.

## ETHICS STATEMENT

Ethical approval for the study was obtained by the West of Scotland Research Ethics Committee 5 (Reference: 20/WS/006).

## Data Availability

Data from the qualitative review (stage 4) are available on request from the first author.
